# G-Protein Coupled Receptor 1 Is Involved in Tetrachlorobisphenol A-Induced Inflammatory Response in Jurkat Cells

**DOI:** 10.3390/toxics12070485

**Published:** 2024-07-02

**Authors:** Xiaoyu Lu, Mengjie Yu, Yingxin Yang, Xiaolan Zhang, Tian Chen, Bingli Lei

**Affiliations:** 1 Institute of Environmental Pollution and Health, School of Environmental and Chemical Engineering, Shanghai University, Shanghai 200444, China; 18221160163@163.com (X.L.); yu15188241737@163.com (M.Y.); yangyingxin2021@163.com (Y.Y.); zhangxiaolan@shu.edu.cn (X.Z.); 2 Department of Environmental Health, Shanghai Municipal Center for Disease Control and Prevention, Shanghai 200336, China; 3 State Environmental Protection Key Laboratory of the Assessment of Effects of Emerging Pollutants on Environmental and Human Health, Shanghai Municipal Center for Disease Control and Prevention, Shanghai 200336, China; 4 NMPA Key Laboratory for Monitoring and Evaluation of Cosmetics, Shanghai Municipal Center for Disease Control and Prevention, Shanghai 200336, China

**Keywords:** tetrachlorobisphenol A, inflammatory response, G-protein coupled receptor 1, molecular mechanism, Jurkat cells

## Abstract

Estrogens can affect the immune inflammatory response through estrogen receptor alpha (ERα), but the specific role of estrogen member receptor G-protein coupled receptor 1 (GPER1) in this process remains unclear. In this study, we evaluated the effects of tetrachlorobisphenol A (TCBPA), which has estrogen activity, on immune inflammatory-related indicators of Jurkat cells, as well as investigated the role of GPER1 in these effects. The results showed that TCBPA at lower concentrations significantly promoted the viability of Jurkat cells, whereas higher concentrations decreased cell viability. TCBPA at concentrations ranging from 1 to 25 μM increased the intracellular reactive oxygen species (ROS) levels. Additionally, treatment with 10 μM TCBPA increased the protein expression of ERα and GPER1, elevated the phosphorylation of protein kinase B (p-Akt), and upregulated the mRNA levels of GPER1, Akt, and phosphoinositide 3-kinase (PI3K) genes. Treatment with 10 μM TCBPA also upregulated the protein or gene expression of pro-inflammatory cytokines, such as interleukins (IL1β, IL2, IL6, IL8, IL12α) and tumor necrosis factor alpha (TNFα) in Jurkat cells. Furthermore, pretreatment with a GPER1 inhibitor G15 significantly reduced the mRNA levels of Akt induced by 10 μM TCBPA. Moreover, the upregulation of mRNA expression of RelA (p65), TNFα, IL6, IL8, and IL12α induced by 10 μM TCBPA was also significantly attenuated after G15 pretreatment. These findings suggest that TCBPA upregulates the expression of genes related to inflammatory responses by activating the GPER1-mediated PI3K/Akt signaling pathway. This study provides new insights into the mechanism of TCBPA-induced inflammatory response.

## 1. Introduction

Tetrachlorobisphenol A (TCBPA), as a chlorinated derivative of bisphenol (BPA), has been detected in various environmental media, including sediment, surface water [1], indoor dust [2], food packaging materials [3], and even in drinking water [4]. There is widespread exposure to the human body. TCBPA has been found in urine [5], blood [6], adipose tissue [7], breast milk [8], and even in embryos [9]. Exposure to TCBPA can lead to changes in the levels of testosterone, estradiol (E_2_), luteinizing hormone, and follicle stimulating hormone, as well as abnormal expression of androgen receptor (AR) genes in the testes, ultimately resulting in reproductive toxicity [10]. Furthermore, TCBPA has demonstrated neurodevelopmental toxicity [11] and genetic toxicity [12,13]. Studies suggest that TCBPA exhibits greater biological lethality and easier bioaccumulation than BPA [14,15]. These studies imply that TCBPA may pose higher health risks than BPA.

TCBPA exhibits similar estrogenic activity to BPA [16]. It can disrupt the functioning of the endocrine system through estrogen nuclear receptor alpha (ERα) or G protein coupled estrogen receptor 1 (GPER1) pathways [17]. The endocrine system is closely related to the immune system, which is considered a natural target of estrogen action [18]. It has been reported that hormones involved in the regulation of endocrine function also mediate inflammatory response [19]. Inflammatory response serves as an important indicator of immune toxicity, and changes in pro-inflammatory cytokines are commonly used to evaluate the occurrence of inflammatory response. Several studies have reported that exogenous estrogens can regulate inflammatory response-related indicators. For instance, Xu et al. [20] found that exposure to BPA disrupts the number and function of innate and adaptive immune cells, leading to decreased levels of Treg cells, anti-inflammatory cytokines, and chemokines, as well as increased production of pro-inflammatory cytokines. Li et al. [21] found that BPA upregulates the expression of pro-inflammatory cytokines in macrophages and increases the production of pro-inflammatory mediators and prostaglandins. Zhao et al. [22] also showed that exposure to bisphenol S (BPS) activates the expression of pro-inflammatory cytokines, such as tumor necrosis factor alpha (TNFα), interleukin-1βeta (IL1β), and IL6, which in turn regulate metabolic pathways and induce pro-inflammatory phenotypes. Wang et al. [23] observed that TCBPA induces immunosuppression and uterine injury in mice, reduces the ratio of CD_3_+T lymphocytes in regulatory T cells, and increases the secretion of four pro-inflammatory cytokines, including IL2, IL12, interferon gamma (IFNγ), and TNFα in mouse serum.

Estrogen receptors are widely distributed in immune organs and immune cells. Therefore, some researchers believe that environmental estrogens may have similar effects on immune cells as they do on endocrine cells. It is believed that these estrogens may mediate changes in immune inflammatory indicators through the ER pathway [24]. Studies have found that nonylphenol (NP) can induce pro-inflammatory responses in sturgeon macrophages by interacting with ERα and nuclear transcription factor nuclear factor (NF-κB) [25]. However, exposure to NP and octyl-phenol (OP) leads to inhibition of Th1 development and enhancement of Th2 development, which is independent of ERs [24]. This indicates that the toxic mechanism of exogenous estrogens on the immune system is complex and not limited only to nuclear receptors. It may also involve the estrogen membrane receptor GPER1. Cai et al. [26] found that GPER1 can stimulate the expression of the classic inflammatory cytokines, such as TNFα and monocyte chemotactic protein-1 (MCP-1). Tang et al. [27] found that GPER1 can mediate aldosterone-induced partial endothelial inflammatory response, possibly through the activation of phosphoinositide 3-kinase (PI3K) signaling pathway. Our previous study found that TCBPA can activate extracellular regulated kinase 1/2 (Erk1/2) and PI3K/protein kinase B (PI3K/Akt) signals through GPER1. This activation leads to the activation of downstream nuclear transcription factors c-Fos, c-Myc, and Cyclin D1, ultimately inducing cell proliferation and changes in intracellular reactive oxygen species (ROS) levels [28]. Therefore, we speculate that the toxic mechanism of TCBPA on the immune system may be related to the activation of GPER1, although the specific details are still unclear.

In this study, Jurkat cells derived from human peripheral blood T lymphocytes were used as a cellular model. We investigated the effects of TCBPA on inflammatory-related indicators. These indicators include cell activity, intracellular ROS levels, and the expression of inflammatory-related proteins and genes. Furthermore, to study the role of GPER1 in TCBPA-induced immune inflammatory response, we employed GPER1 inhibitor G15 to examine the regulation of PI3K/Akt signaling pathway mediated by GPER1 on the inflammatory-related indicators. This study will contribute to providing a novel understanding of the immunomodulation mechanism of TCBPA. Additionally, it may offer a theoretical foundation for analyzing the relationship between TCBPA exposure and the occurrence of immune inflammatory-related diseases.

## 2. Materials and Methods

### 2.1. Reagents

Tetrachlorobisphenol A (TCBPA) (Cas No. 79-95-8, 98%) was purchased from Sigma-Aldrich (Saint Louis, MO, USA). It was dissolved in dimethyl sulfoxide (DMSO, Cas No. 67-68-5, 99.9%) and stored at −20 °C. Quantities of 2′,7′-dichlorodihydrofluorescein diacetate (DCFH-DA, Cas No. 4091-99-0, ≥95%), ter-butyl hydroperoxide (t-BHP, Cas No. 75-91-2, 98%), and CCK-8 were also obtained from Sigma-Aldrich (Saint Louis, MO, USA). The SYBR Green Dye and the reverse transcription kit were purchased from TOYOBO (Osaka, Japan). Additionally, all other reagents used in this study were of analytical grade, unless otherwise specified.

### 2.2. Maintance and Treatment

Jurkat cells (source: American Type Culture Collection, ATCC) from human peripheral blood T lymphocytes were purchased from Yuchi Biotechnology Co., Ltd. in Shanghai, China. Jurkat cells were incubated in RPMI-1640 culture medium supplemented with 10% fetal bovine serum (FBS, Gibco) at 37 °C in a cell culture incubator containing 5% CO_2_. The logarithmic growth of Jurkat cells was used in the exposure experiment. A quantity of 0.1% DMSO was used as the negative control.

### 2.3. Cell Viability

CCK-8 colorimetric assay is commonly used to evaluate cell viability because of its sensitivity and operational convenience [29]. In this study, Jurkat cells in the logarithmic growth phase at the density of 50,000–100,000/mL were adjusted in RPMI-1640 low glucose culture medium containing 1000 mg/L glucose. The 100 μL of cell suspension containing 0.01–100 μM TCBPA or 0.1% DMSO was added to each well of a 96-well culture plate. For each treatment, five parallel samples were prepared. The cells were then exposed for 24, 48, and 72 h. After treatment, 10 μL of CCK-8 solution was added to each well. After incubating for 4 h, the absorbance (OD) of each well was measured at 450 nm using a spectrophotometer. Cell viability was calculated by comparing the OD values of treatment groups with those of the control group.

### 2.4. Detection of Intracellular Reactive Oxygen Species (ROS) Levels

In this study, intracellular ROS levels are detected using a nonpolar dye DCFH-DA probe. The DCFH-DA probe is able to penetrate the cell membrane and be converted into the polar derivative dichlorodihydrofluorescein (DCFH) by intracellular esterases [30]. DCFH does not exhibit fluorescence, but it can be oxidized by intracellular ROS into the highly fluorescent dichlorofluorescein (DCF). Therefore, the fluorescence intensity of DCF can be used to reflect the levels of intracellular ROS. Jurkat cells in the logarithmic phase with a density of 100,000 to 150,000 cells/mL were resuspended in RPMI-1640 low glucose culture medium. The 1 mL of cell suspension per well, containing 0.1–25 μM TCBPA or 0.1% DMSO, was inoculated into a 6-well culture plate. The cells were exposed for 24 h. One hour before the end of exposure, untreated positive control cells were collected and resuspended in RPMI-1640 low glucose culture medium containing 200 μM t-BHP. After treatment, Jurkat cells were resuspended in the diluted DCFH-DA solution. The cells were then incubated in a dark incubator at 37 °C for approximately 30 min, with occasional inversion every 3–5 min to ensure sufficient contact between the cells and the probe. After incubation, the cells were centrifuged and washed three times to remove any residual culture medium containing DCFH-DA that has not entered the cells. The cells were then resuspended in 500 μL D-Hank’s buffer, and the fluorescence intensity was measured at excitation wavelength 488 nm and emission wavelength 525 nm using flow cytometry (Beckman, CA, USA).

### 2.5. Western Blot for Protein Detection

Western blot was used to detect protein expression in Jurkat cells in this study. The specific protocol for Western blot can be found in our previous study [31]. Briefly, the nuclear protein of ERa and total protein samples of other targets were obtained by NE-PER and M-PER, respectively. Equal amounts (20 mg) of protein samples were subjected to 10% sodium dodecyl sulfate-polyacrylamide gel electrophoresis (SDS-PAGE) and then transferred to a solid phase carrier PVDF membrane. The membranes were blocked in a 5% milk containing TNT buffer. The membranes were then incubated in turn with primary and secondary antibodies for 1 h at room temperature in a constant speed oscillator. The target protein on the membrane can bind specifically to the corresponding primary antibody, and then reacts with the secondary antibody labeled by horseradish peroxidase (HRP). Finally, target protein bands were visualized using imaging technology. In this study, the specific mono/polyclonal antibodies used were anti-ERα (1:1000), anti-GPER1 (1:400), anti-Akt (1:1000), anti-p-Akt (1:2000), anti-TNFα (1:1000), anti-IL1β (1:1000), anti-IL2 (1:1000), anti-IL6 (1:1000), and anti-GAPDH (1:10,000). GAPDH was used as a reference.

### 2.6. RT-qPCR for mRNA

RT-qPCR technology was used to detect the expression of target genes. Jurkat cells in logarithmic growth phase were resuspended in RPMI-1640 low glucose culture medium containing different concentrations of TCBPA. The cells were then inoculated into a six-well plate for 12 h. After treatment, the cells were lysed, and total RNA was extracted and purified using the same method as described in our previous study [28]. The A260/A280 ratios of all samples ranged from 1.81 to 2.18 and the A260/A230 ratios of all samples ranged from 2.05–2.52, indicating that the extracted RNA was of good quality for further reverse-transcriptase polymerase chain reaction (RT-PCR). The purified RNA was used to perform RT-PCR in order to obtain cDNA template for subsequent second-strand synthesis and amplification, following the instructions provided in the manual of the reverse transcription kit (ReverTra Ace^®^, TOYOBO. Co., Ltd., Osaka, Japan). The reverse transcription reaction solution (10 μL) contained 2.0 μL of RT buffer, 0.5 μL of primer mix, 0.5 μL of enzyme mix, and 7 μL of nuclease free water and isolated RNA (1000 ng). The PCR amplification was performed using the ABI 7500 fast system (ABI, MA, USA), according to the following protocol: pre-denaturation at 95 °C for 60 s, followed by 50 cycles of denaturation at 95 °C for 15 s, annealing at 60 °C for 15 s, extension at 72 °C for 45 s, and end extension at 65 °C for 5 s. The dissolution curve analysis was performed with a temperature range of 65 °C to 95 °C, with a temperature increase of 0.5 °C every 5 s. In subsequent gene expression analysis, the housekeeping gene β-actin was used as the internal reference gene. The relative mRNA levels of the target genes were calculated using the following equation: R = 2^−ΔΔCt^ [32] (Lei et al. 2021b). Specific primers for the target genes were obtained from Wcgene Biotech (Shanghai, China), and the primer sequences are listed in Table S1 in Supporting Information.

### 2.7. Data Analysis

All experiments were repeated at least twice to ensure the reliability of the results. The data are presented as mean ± standard deviation (S.D.) based on representative results. Statistical analyses were performed using SPSS ver.19.0 (SPSS, Chicago, IL, USA). Differences between the TCBPA treatment groups and the DMSO control group were evaluated using one-way analyses of variance (ANOVA), followed by specific mean comparisons using Dunnett’s test. Differences between two exposure groups were determined by a Student’s *t*-test. A *p*-value less than 0.05 was considered statistically significant.

## 3. Results

### 3.1. Effect of TCBPA on the Viability of Jurkat Cells

**Figure 1:**
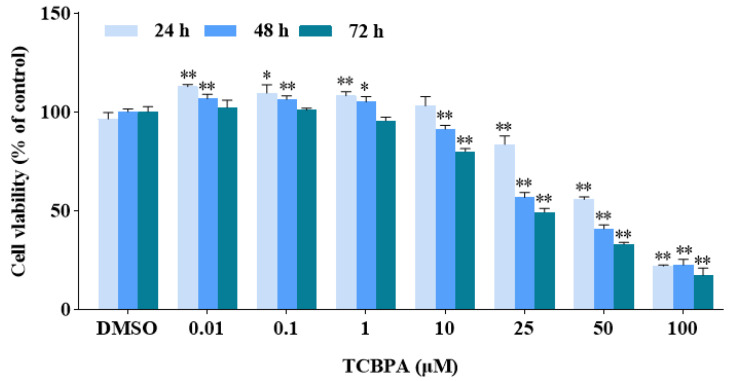
The effect of TCBPA on the viability of Jurkat cells. The experiment was repeated three times with 5 parallel samples for each time. The results are shown based on the mean ± standard deviation (S.D.) of 5 parallel samples from a representative result. * *p* < 0.05 and ** *p* < 0.01 compared to 0.1% DMSO.

The effects of TCBPA (0.01–100 μM) on the viability of Jurkat cells are shown in Figure 1. TCBPA at 0.01–1 μM and exposure for 24 and 48 h significantly increased the viability of Jurkat cells. However, exposure to TCBPA at 10–25 μM significantly inhibited the cell viability in a time- and concentration-dependent manner.

### 3.2. The Effect of TCBPA on the Intracellular ROS Levels

**Figure 2:**
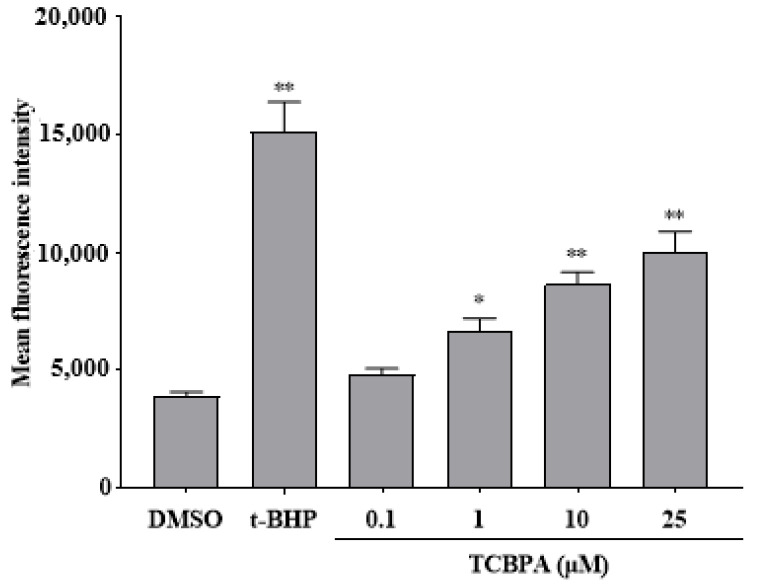
The effect of TCBPA on intracellular ROS levels in Jurkat cells. The experiment was repeated twice with 3 parallel samples for each time. The results are shown based on the mean ± S.D. of three parallel samples from a representative result. * *p* < 0.05 and ** *p* < 0.01 compared to 0.1% DMSO.

As shown in Figure 2, the fluorescence intensity of Jurkat cells treated with TCBPA was used to assess changes in intracellular ROS levels. The results showed that TCBPA at 1–25 μM resulted in a significant concentration-dependent increase in the fluorescence intensity of cells. Notably, at 25 μM, the fluorescence intensity was the highest, indicating the most substantial increase in ROS levels.

### 3.3. The Effect of TCBPA on the Expression of Target Proteins Associated with Estrogen Receptor-and Inflammatory-Related Targets

**Figure 3:**
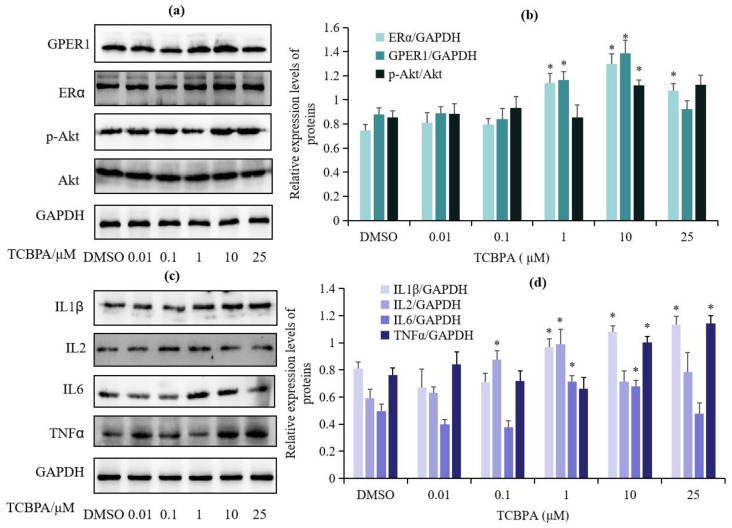
The effect of TCBPA on the expression of estrogen receptor- and inflammatory-related proteins in Jurkat cells. (**a**) The effects of TCBPA on the expression of GPER1, ERα, and p-Akt proteins; (**b**) grey-level quantification values of target protein bands of GPER1, ERα, and p-Akt; (**c**) the effect of TCBPA on the expression of IL-1 β, IL2, IL6, and TNFα proteins; (**d**) grey-level quantification values of target protein bands of IL-1 β, IL2, IL6, and TNFα. The experiment was repeated twice with two parallel samples for each time. The results are shown based on the mean ± S.D. of two parallel samples. * *p* < 0.05 compared to 0.1% DMSO.

In order to detect whether estrogen receptors and related signals are activated after exposing Jurkat cells to TCBPA, we selected the concentrations of TCBPA that resulted in Jurkat cell viability over 80% as the exposure concentrations for protein expression. Jurkat cells were exposed to TCBPA at concentrations of 0.01, 0.1, 1, 10, and 25 μM for 24 h, and the expression of estrogen signaling pathway-related proteins, including GPER1, p-Akt, and ERα, is shown in Figure 3a,b. TCBPA at concentrations of 1–25 μM significantly increased the expression of ERα protein, while the concentrations of 1–10 μM upregulated the expression of GPER1 protein. The phosphorylation of Akt was significantly upregulated only at a concentration of 10 μM TCBPA. The results indicate that TCBPA activates estrogen receptor-related targets in Jurkat cells.

In order to investigate the regulation of GPER1 activation on inflammatory-related targets after exposure to TCBPA, the protein expression of typical pro-inflammatory cytokines such as IL1β, IL2, IL6, and TNFα was also detected. As shown in Figure 3c,d, TCBPA at 1–25 μM significantly upregulated the protein expression of IL1β, while at concentrations of 0.1 and 1 μM, it upregulated the expression of IL2 protein. Furthermore, TCBPA at concentrations of 1 and 10 μM significantly upregulated the protein expression of IL6, and at concentrations of 10 and 25 μM, it upregulated the protein expression of TNFα. These results indicate that under current exposure conditions, TCBPA changed the expression of inflammatory-related proteins.

### 3.4. The Effect of TCBPA on the Expression of Inflammatory Response-Related Genes

**Figure 4:**
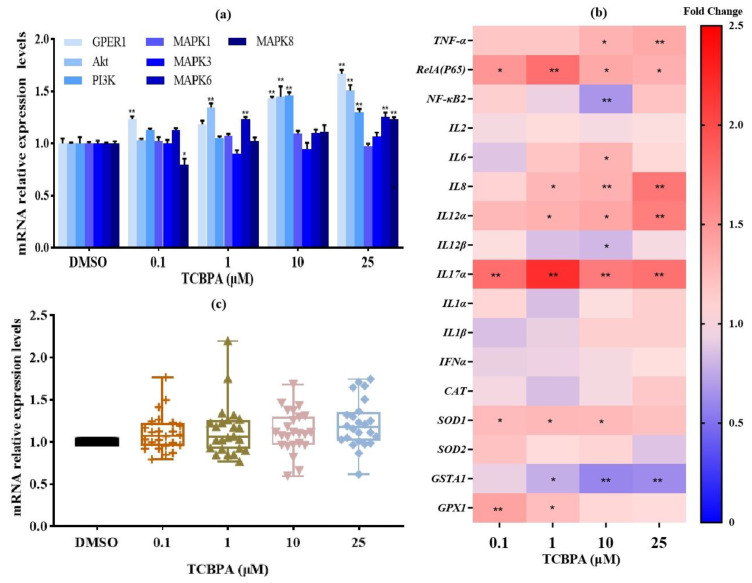
The effect of TCBPA on the expression of 24 target genes in Jurkat cells. (**a**) Histogram of mRNA expression of 7 target genes related to estrogen signaling pathway; (**b**) heat map of mRNA expression of 17 target genes related to antioxidant and inflammatory responses; (**c**) the dispersion of data on the mRNA levels of 24 target genes. The experiment was repeated twice with three parallel samples for each time. The data are represented as the mean ± S.D. of three parallel samples from a representative result. * *p* < 0.05 and ** *p* < 0.01 compared to 0.1% DMSO.

The effects of TCBPA on the expression of target genes were also investigated. As shown in Figure 4a,b, exposure to different concentrations of TCBPA significantly altered the mRNA levels of 16 target genes, with most of them being upregulated. The largest number of target genes was affected by a concentration of 10 μM TCBPA. Specifically, TCBPA at 10 μM significantly upregulated the expression of three estrogen receptor signaling-related genes (GPER1, PI3K and Akt), as well as seven inflammatory-related genes (RelA (p65), IL6, IL8, IL12α, IL17α, TNFα, and superoxide dismutase 1 (SOD1)). Additionally, TCBPA significantly downregulated the mRNA expression of NF-κB_2_, IL12β, and glutathione S-transferase alpha 1 (GSTA1). There was no significant change in mRNA levels of mitogen-activated protein kinase 1 (MAPK1), MAPK3, IL1α, IL1β, IL2, INFα, catalase (CAT), and SOD_2_ at any of the test concentrations. The relative mRNA values of most genes under different exposure concentrations of TCBPA were distributed between 0.5 and 2.0 (Figure 4c). This indicated that the mRNA values of these target genes are relatively tightly clustered under different exposure concentrations of TCBPA.

### 3.5. Regulation of GPER1 on the Expression of Pro-Inflammatory-Related Genes

**Figure 5:**
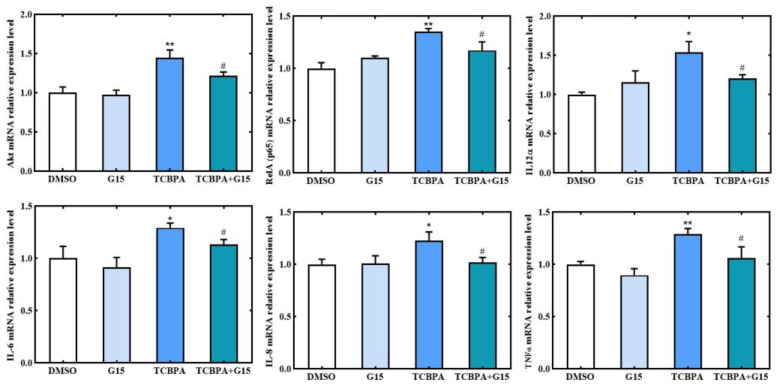
The effect of GPER1 inhibitor G15 on the expression of Akt and inflammation-related target genes induced by TCBPA in Jurkat cells. Jurkat cells were pre-treated with G15 (10 µM) for 1 h and then treated with TCBPA (10 µM) for 12 h. The experiment was repeated twice with three parallel samples for each time. The data are represented as mean ± S.D. of three parallel samples from a representative result. * *p* < 0.05, ** *p* < 0.01 compared to 0.1% DMSO by Dunnett’s test; ^#^ *p* < 0.05, compared to 10 TCBPA alone treatment by Student’s *t*-test.

TCBPA is a very important halogenated derivative of BPA and has estrogenic activity [16]. Exposure to TCBPA resulted in upregulation of both GPER1 protein and gene expression, as well as pro-inflammatory-related proteins and genes in Jurkat cells. Therefore, we conducted further investigations to determine whether TCBPA-induced inflammatory response is associated with the activation of GPER1. Among the target proteins and genes affected by TCBPA, the highest number of changes was observed with a concentration of 10 μM. Therefore, for the subsequent inhibitor experiment, 10 µM was set as exposure concentration of TCBPA. To examine the effects of GPER1 activation on the expression of inflammatory-related genes induced by 10 µM TCBPA, we employed G15, a GPER1 inhibitor. Pretreatment with G15 (10 μM) for 1 h significantly inhibited the upregulation of mRNA expression for Rela (p65), IL12α, IL6, IL8, and TNFα induced by TCBPA, as shown in Figure 5. Furthermore, G15 pretreatment also significantly inhibited the upregulation of Akt mRNA expression induced by TCBPA.

## 4. Discussion

In this study, we found that exposure to TCBPA led to the expression upregulation of inflammatory-related proteins or genes, including ILs, TNFα, and NF-κB. ILs are very important members of cytokine family, with IL1β, IL2, IL6, IL8, and IL12α being particularly important pro-inflammatory cytokines [33]. These cytokines play critical roles in mediating cellular immunity, and alterations in their expression are closely related to the various immunity-related diseases [34]. Our findings revealed a significant upregulation in the expression of IL1β, IL2, IL6, and IL8 proteins and/or genes upon TCBPA exposure. A similar phenomenon has been observed for other bisphenol compounds. For instance, BPA has been shown to dose-dependently promote the production of IL1β and IL6 in placental explant [35], and nanomolar concentrations of BPA increased IL6 expression [36]. BPA also stimulates the expression of IL1β in human mast cells (HMC-1) [37]. Zhang et al. [38] found that BPF exposure increased secretion of pro-inflammatory cytokine IL1β in RAW264.7 macrophages. Wang et al. [23] reported that TCBPA exposure significantly induced an increase in the secretion of the pro-inflammatory cytokine IL2 in mouse serum. Russo et al. [39] demonstrated that exposure to BPA for 24 h markedly upregulated the expression of IL1β and IL8 genes of human umbilical vein endothelial cells.

We also observed that TCBPA exposure at concentrations of 10 and 25 µM upregulated the expression of TNFα protein and gene. TNFα is a crucial pro-inflammatory cytokine and can induce the production of cytokines such as IL1, IL6, and IL8, activate multiple signal transduction pathways, kinases, and transcription factors, and upregulate the expression of numerous cellular genes [40]. Additionally, in this study, we found that TCBPA at 10 µM increased the mRNA levels of IL6 and IL8 genes. Moon et al. [41] demonstrated that BPA induced the production of pro-inflammatory cytokines, including IL6 and TNFα, in mouse liver. BPA also leads to the upregulation of the TNFα protein expression, which mediates pulmonary inflammatory response and significantly increases the expression of the TNFα gene [42].

In addition, in this study, TCBPA at 0.1–25 μM significantly upregulated the mRNA levels of RelA (p65) and IL17α. RelA (p65) is one member of NF-κB family. NF-κB is a homo- or heterodimeric complex formed by the Rel-like domain-containing proteins, including RelA (p65), RelB, c-Rel, NF-κB1(p50), and NF-κB2 (p52) [43]. Improper activation of NF-κB has been associated with inflammatory events related to autoimmune arthritis, asthma, lung fibrosis, glomerulonephritis, and atherosclerosis [43]. RelA has also been shown to modulate immune responses, and its activation is positively associated with multiple types of cancer [44]. Akt is capable of phosphorylating RelA under different conditions, and this phosphorylation plays a crucial role in regulating NF-κB activation and function [45]. In this study, we also found that TCBPA at concentrations of 1–25 μM upregulated the mRNA levels of Akt and RelA (p65) genes. IL17α, as IL17 subunit, is widely recognized as a pathogenic cytokine. Wang et al. [46] found that IL17α exacerbates middle ear injury by upregulation of IL6, TNFα, and myeloperoxidase expression. This study also found that exposure to TCBPA upregulated the gene and protein expression of IL6 and TNFα and increased the expression of IL17α gene. It is possible that TCBPA induces the production of IL17α, which further upregulates the expression of IL6 and TNFα.

Our previous study found that TCBPA exhibits estrogenic activity and can stimulate the proliferation of MCF-7 estrogen-sensitive tumor cells by activating ERα and GPER1 signaling pathways [28]. In this study, we also observed that TCBPA at 1 μM upregulated the expression of ERα and GPER1 proteins and enhanced cell viability. This indicates that TCBPA activates the estrogen receptor signaling pathways in Jurkat cells.

ERα is a typical nuclear estrogen receptor which has a close relationship between the activation of ERα signaling pathway and the occurrence of inflammation and relevant diseases [47,48]. In addition, as a novel estrogen membrane receptor, GPER1 also play an important role in mediating immune-related diseases, such as multiple sclerosis, Parkinson’s disease and atherosclerosis-related inflammation [49]. G1, as GPER1 agonist, has been shown to dose-dependently increase the release of pro-inflammatory cytokine IL8 from human neutrophils at the concentrations of 10 and 100 µM [50]. Notas et al. [49] even proposed that GPER1 appears to play a critical role in the functional regulation of all immune cells and several pro-inflammatory mechanisms. In recent years, the involvement of GPER1 in mediating both the pro-inflammatory and anti-inflammatory actions of estrogens has gained increasing concern. For example, Cai et al. [26] showed that GPER1 contributes to the regulation of estrogen-induced inflammation, and its activation can upregulate the expression of the typical inflammatory cytokine TNFα and MCP-1. Li et al. [51] revealed that BPA can promote the progression of laryngeal cancer by upregulating IL6 via GPER. In the present study, we observed that TCBPA at a concentration of 10 μM increased the protein expression of GPER1 and ERα. Moreover, the protein expression of inflammatory cytokines such as IL1β, IL6, and TNFα was also elevated. These findings suggest that GPER1 might also play a crucial role in the TCBPA-induced inflammatory response.

PI3K/Akt signaling pathway plays a critical role in numerous cellular biological processes. Zhang et al. [38] discovered that BPF can enhance glycolysis through the PI3K/Akt signaling pathway, thereby promoting the secretion of pro-inflammatory cytokines. Gao et al. [52] showed that perinatal exposure to BPA leads to an imbalance of Treg/Th17 cells in male offspring mice by activating the PI3K/Akt/mTOR signaling pathway. Furthermore, Guo et al. [53] found that low molecular weight PAHs activate PI3K/Akt and NF-κB signaling pathways, inducing an inflammatory response in A549 cells. PI3K/Akt pathway is a well-known non-genomic signaling pathway regulated by GPER1 [28]. GPER1 can mediate partial aldosterone that induces endothelial inflammatory response, which may be related to the activation of the PI3K signaling pathway [27,54]. In this study, we also found that TCBPA at a concentration of 10 μM increased the protein expression of PI3K, p-Akt and GPER1, and upregulated the mRNA levels of Akt and GPER1 genes, as well as genes related to inflammatory response. The GPER1 inhibitor G15 reversed the mRNA levels of Akt induced by TCBPA, indicating that GPER1 regulates the PI3K/Akt signaling pathway. Additionally, G15 attenuated the expression of pro-inflammatory cytokines such as TNFα, RelA (p65), IL12A, IL8, and IL6 genes induced by TCBPA, suggesting that GPER1 activation by TCBPA leads to expression upregulation of inflammatory-response-related genes.

In addition, there is a close relationship between oxidative stress and inflammatory response. Tantengco et al. [55] found that oxidative stress can promote inflammation in cervical cells. Oxidative stress refers to an imbalance between oxidative and antioxidant effects in the body, and it can be directly evaluated by measuring intracellular ROS levels [56]. Therefore, ROS plays a crucial role as a signaling molecule and inflammatory mediator in various inflammation-related diseases [57]. When cells experience oxidative stress, numerous essential endogenous antioxidant enzymes, such as glutathione peroxidase (GPX), SOD, glutathione S-transferase (GST), and glutathione (GSH), are activated to eliminate oxidant molecules like free radicals and superoxide anions, thus maintaining the redox balance of cells to the greatest extent possible [58,59]. The expression of genes related to antioxidants can be regulated by antioxidant enzyme activity. Hence, the expression of antioxidant-related genes can partially reflect the body’s antioxidant capacity [12]. In this study, TCBPA at concentrations of 0.1–1 or 10 μM upregulated the expression of SOD1 and GPX1 genes, indicating that Jurkat cells undergo oxidative stress. Additionally, TCBPA at concentrations of 1–25 µM downregulated the gene expression of GSTA1, suggesting that TCBPA may inhibit the activity of antioxidant enzyme GSTA1. Shumilla et al. [60] proposed that a decrease in GST activity after exposure to heavy metals could be attributed to the direct action of metals on the enzyme or inhibition of GST by excessive ROS. In our study, we also found that TCBPA at concentrations of 1–25 µM significantly increased intracellular ROS levels. These findings indicate that TCBPA induces oxidative stress in cells, thereby reducing the antioxidant capacity of Jurkat cells.

**Figure 6:**
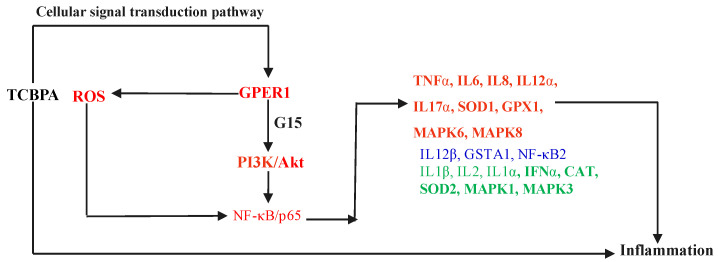
The proposed cellular signal transduction pathway of TCBPA-induced cell inflammation in Jurkat cells. All genes in the figure were detected in this study. Red indicates a significant upregulation, green indicates no obvious change and blue indicates a significant downregulation, compared with 0.1% DMSO.

Furthermore, several studies have shown that intracellular ROS generation induced by low concentrations of environmental estrogens is closely linked to the activation of estrogen receptors. For example, treatment to low concentrations of BPA can increase intracellular ROS levels through ERα activation in GT1-7 cells [61]. Kim et al. [62] discovered that an increase in ROS may be associated with the activation of GPER1 signaling, leading to Akt phosphorylation. In our previous study, we also found that low concentrations of BPAF increase the intracellular ROS levels by activating GPER1-mediated PI3K/Akt signaling pathway [56]. In the present study, we observed significant upregulation of intracellular ROS levels and GPER1 expression at an exposure concentration of 10 μM TCBPA. Additionally, at this concentration, the gene or protein expression of certain pro-inflammatory cytokines such as IL1β, IL6, IL8, IL12α, and TNFα was also significantly upregulated. These results suggest that ROS may also be involved in the occurrence of inflammatory response induced by TCBPA and may be related to GPER1 activation. Based on the findings from this study and previously published literatures, we proposed a cellular signal transduction pathway for TCBPA-induced cell inflammation in Jurkat cells (Figure 6).

## 5. Conclusions

The results showed that high concentrations of TCBPA significantly inhibited cell viability, while low concentrations of TCBPA increased cell viability. TCBPA upregulated the expression of estrogen receptor- and inflammatory-related protein or genes. Through inhibitor experiments, we found that GPER1 plays an important role in regulating the expression of inflammatory-related genes. Additionally, TCBPA induced the generation of intracellular ROS and altered the expression of antioxidant enzyme genes such as GSTA1, SOD1, and GPX1, indicating that TCBPA induces oxidative stress and affects the antioxidant capacity of Jurkat cells. These findings are consistent with the expression of inflammatory response-related proteins or genes induced by TCBPA, suggesting that ROS may also be involved in the occurrence of inflammatory response induced by TCBPA. These findings provide new insights into the mechanism of TCBPA-induced inflammatory response.

## Data Availability

The data that support the findings of this study are available from the corresponding author upon reasonable request.

## Supplementary Materials

The following supporting information can be downloaded at: https://www.mdpi.com/article/10.3390/toxics12070485/s1, Table S1: The primer sequences of target genes.
