# Supplementary material for: G-Protein Coupled Receptor 1 Is Involved in Tetrachlorobisphenol A-Induced Inflammatory Response in Jurkat Cells
Source: Toxics. 2024 Jul 2;12(7):485. doi: 10.3390/toxics12070485 (PMC11281156; doi:10.3390/toxics12070485)
Supplement: Supplementary file 1 [file toxics-12-00485-s001.zip › toxics-3054874-supplementary.pdf]

G-protein Coupled Receptor 1 Is Involved in Tetrachlorobisphenol A-Induced Inflammatory Response in Jurkat Cells

Xiaoyu Lu [1,†], Mengjie Yu [1,†], Yingxin Yang [1], Xiaolan Zhang [1], Tian Chen [2,3,4,*] and Bingli Lei [1,*]

[1] Institute of Environmental Pollution and Health, School of Environmental and Chemical Engineering, Shanghai University, Shanghai 200444, China; 18221160163@163.com (X.L.); yu15188241737@163.com (M.Y.); yangyingxin2021@163.com (Y.Y.); zhangxiaolan@shu.edu.cn (X.Z.)

[2] Department of Environmental Health, Shanghai Municipal Center for Disease Control and Prevention, Shanghai 200336, China

[3] State Environmental Protection Key Laboratory of the Assessment of Effects of Emerging Pollutants on Environmental and Human Health, Shanghai Municipal Center for Disease Control and Prevention, Shanghai 200336, China

[4] NMPA Key Laboratory for Monitoring and Evaluation of Cosmetics, Shanghai Municipal Center for Disease Control and Prevention, Shanghai 200336, China

* Correspondence: chentian@scdc.sh.cn (T.C.); leibingli@126.com (B.L.); Tel.: +86-21-66137734 (B.L.)

† These authors contributed equally to this work.

Table S1 The primer sequences of target genes

Genes	F primer	R primer
GPER1	CCTGCTTCTGTTTCGCGGAT	CAATGAGGGAGTAGCACAGGC
Akt	ACCTTCCATGTGGAGACTCCTGAG	GTCCATCTCCTCCTCCTCCTGC
PI3K	AGAGCACTTGGTAATCGGAGG	CTTCCCCGGCAGTATGCTTC
MAPK1	TCACACAGGGTTCCTGACAGA	ATGCAGCCTACAGACCAAATATC
MAPK3	ATGTCATCGGCATCCGAGAC	GGATCTGGTAGAGGAAGTAGCA
MAPK6	TGTCAAACATGCTCTACGTGAAA	TCGTCTGTTAATTGGCTTCCAC
MAPK8	TCTGGTATGATCCTTCTGAAGCA	TCCTCCAAGTCCATAACTTCCTT
TNFα	GGCCCGACTATCTCGACTTTG	CGTTTGGGAAGGTTGGATGTT
RelA(p65)	CCCACGAGCTTGTAGGAAAGG	GGATTCCCAGGTTCTGGAAAC
NF-κB2	AGAGGCTTCCGATTTCGATATGG	GGATAGGTCTTTCGGCCCTTC
IL2	CCCAAGAAGGCCACAGAACT	GCACTTCCTCCAGAGGTTTGA
IL6	CCTGAACCTTCCAAAGATGGC	TTCACCAGGCAAGTCTCCTCA
IL8	TTTTGCCAAGGAGTGCTAAAGA	AACCCTCTGCACCCAGTTTTC
IL12α	GCACAGTGGAGGCCTGTTTA	GGATAGGTCTTTCGGCCCTTC
IL12β	GCGGAGCTGCTACACTCTC	GCCAGGCAACTCCCATTAGT
IL17α	AGATTACTACAACCGATCCACCT	GGGGACAGAGTTCATGTGGTA
IL1α	AGATGCCTGAGATACCCAAAACC	CCAAGCACACCCAGTAGTCT
IL1β	ATGATGGCTTATTACAGTGGCAA	GTCGGAGATTCGTAGCTGGA
IFNα	ACTCATACACCAGGTCACGC	CAGTGTAAAGGTGCACATGACG
CAT	TGAAGATGCGGCGAGACTTT	GAGGGGTACTTTCCTGTGGC
SOD1	TAGCGAGTTATGGCGACGAA	CCACACCTTCACTGGTCCAT
SOD2	GGAAGCCATCAAACGTGACTT	CCCGTTCCTTATTGAAACCAAGC
GSTA1	CTCCTTCTGCCCGTATGTCC	TCAAAGGCAGGGAAGTAGCG
GPX1	CAGTCGGTGTATGCCTTCTCG	GAGGGACGCCACATTCTCG
β-Actin	AAACGTGCTGCTGACCGAG	TAGCACAGCCTGGATAGCAAC
